# Human cerebral malaria and *Plasmodium falciparum *genotypes in Malawi

**DOI:** 10.1186/1475-2875-11-35

**Published:** 2012-02-07

**Authors:** Danny A Milner, Jimmy Vareta, Clarissa Valim, Jacqui Montgomery, Rachel F Daniels, Sarah K Volkman, Daniel E Neafsey, Daniel J Park, Stephen F Schaffner, Nira C Mahesh, Kayla G Barnes, David M Rosen, Amanda K Lukens, Daria Van-Tyne, Roger C Wiegand, Pardis C Sabeti, Karl B Seydel, Simon J Glover, Steve Kamiza, Malcolm E Molyneux, Terrie E Taylor, Dyann F Wirth

**Affiliations:** 1 Department of Pathology, Brigham and Women's Hospital, 75 Francis Street, Amory 3, Boston, MA 02115, USA; 2 The Blantyre Malaria Project, University of Malawi College of Medicine, Blantyre, Malawi; 3 Department of Immunology and Infectious Disease, Harvard School of Public Health, Boston, MA 02115, USA; 4 College of Medicine, Malawi-Liverpool-Wellcome Trust Clinical Research Programme, Blantyre, Malawi; 5 Liverpool School of Tropical Medicine, University of Liverpool, Liverpool, UK; 6 Broad Institute, Cambridge, MA 02142, USA; 7 Department of Nursing, School for Health Sciences, Simmons College, Boston, MA 02115, USA; 8 College of Osteopathic Medicine, Michigan State University, East Lansing, MI 48824, USA; 9 College of Medicine, University of Malawi, Blantyre, Malawi; 10 Center for Systems Biology, Department of Organismic and Evolutionary Biology, Harvard University, Cambridge, MA, USA

**Keywords:** *Plasmodium falciparum*, Cerebral malaria, Genotyping, Molecular barcode, Histopathology, Autopsy

## Abstract

**Background:**

Cerebral malaria, a severe form of *Plasmodium falciparum *infection, is an important cause of mortality in sub-Saharan African children. A Taqman 24 Single Nucleotide Polymorphisms (SNP) molecular barcode assay was developed for use in laboratory parasites which estimates genotype number and identifies the predominant genotype.

**Methods:**

The 24 SNP assay was used to determine predominant genotypes in blood and tissues from autopsy and clinical patients with cerebral malaria.

**Results:**

Single genotypes were shared between the peripheral blood, the brain, and other tissues of cerebral malaria patients, while malaria-infected patients who died of non-malarial causes had mixed genetic signatures in tissues examined. Children with retinopathy-positive cerebral malaria had significantly less complex infections than those without retinopathy (OR = 3.7, 95% CI [1.51-9.10]).The complexity of infections significantly decreased over the malaria season in retinopathy-positive patients compared to retinopathy-negative patients.

**Conclusions:**

Cerebral malaria patients harbour a single or small set of predominant parasites; patients with incidental parasitaemia sustain infections involving diverse genotypes. Limited diversity in the peripheral blood of cerebral malaria patients and correlation with tissues supports peripheral blood samples as appropriate for genome-wide association studies of parasite determinants of pathogenicity.

## Background

The global *Plasmodium falciparum *parasite population is highly diverse, especially in antigens transported to the erythrocyte surface where they can interact with the human immune system [1,2]. The major question being addressed, "Are there parasite genetic determinants of cerebral malaria and can we identify them?" requires careful step-wise considerations. Background multiplicity of *Plasmodium falciparum *infection in both asymptomatic and symptomatic individuals is high in Malawi due to intense transmission. Several sequencing approaches using material directly from tissue or peripheral blood have been useful for SNP discovery at the population level. Understanding sequencing data from mixed infections however has been difficult to interpret and quantify for an individual parasite genotype within a single patient. Previously, attempts to evaluate individual *var *gene transcripts from patients by sequencing showed a vast array of clones per patient [3]. Therefore, this study steps back and asks the question at the whole-genome level.

Previously, a 24 marker, single nucleotide polymorphisms (SNP) TaqMan assay (i.e. a molecular barcode) was developed for genotyping *P. falciparum *parasites in the laboratory and it was introduced here for the current field study of clinical samples [4]. The molecular barcode assigns a unique identity to parasite clones, which can be followed during in vitro culture. SNPs, which are fixed mutations in a population, are theoretically not prone (as is the case with multiple repeat regions) to alterations from season to season or during culture adaptation. The molecular barcode is simpler to perform than capillary electrophoresis with *msp-1 *and -*2 *and requires only an RT-PCR device; thus, it is amenable to field deployment. The molecular barcoding is used in the present study: a) to evaluate the performance of the tool in clinical samples which are likely to be a mixture of multiple clones, and b) to understand the possible quantitative proportion data provided by this technique in field samples.

The initial approach was to explore the performance of the molecular barcode in autopsy tissue and blood in patients with retinopathy-positive and retinopathy-negative cerebral malaria, comparing the barcode results with *msp-1 *and -*2 *data [5]. The molecular barcode was then analysed exclusively in the peripheral blood of living patients with clinically defined cerebral malaria, comparing results in patients with and without malarial retinopathy.

Three hypotheses were tested:

1 The *P. falciparum *variant(s) in the peripheral blood mirror those in the tissues (as suggested from indirect evidence [6]). Support for this hypothesis would facilitate studies of malaria pathogenesis because parasites in the peripheral blood are more readily accessible than those in the relevant tissues.

2 The molecular barcode corroborates previously published data suggesting that more severe malaria illnesses are associated with less complex infections [5,7-11]. Important co-factors were included in this analysis, one of these being the date of admission (i.e. time point in the malaria season), since increasing acquisition of immunity during the course of a season might have an effect on clinical infections.

3 Patients with cerebral malaria have a single predominant parasite genotype.

## Methods

### Definition of cerebral malaria

The World Health Organization's (WHO) clinical definition of cerebral malaria (CM) includes the following: a Blantyre coma score ≤ 2, *P. falciparum *parasitaemia by blood film, and no other evident cause of coma (e.g. meningitis, post-ictal state, hypoglycaemia) [12]. The definitive diagnosis of CM relies on post-mortem examination of the brain either by autopsy or supra-orbital sampling. Finding malarial retinopathy on ophthalmoscopy in a comatose paediatric patient supports a diagnosis of CM [13-17]. Patients meeting the clinical case definition, but who do not have malaria retinopathy, are clinically heterogeneous; on autopsy there is very little histological evidence of sequestered parasites in the microvasculature of the brain and other organs, and usually another cause of death is found [18].

### Study design and patients

**Figure 1:**
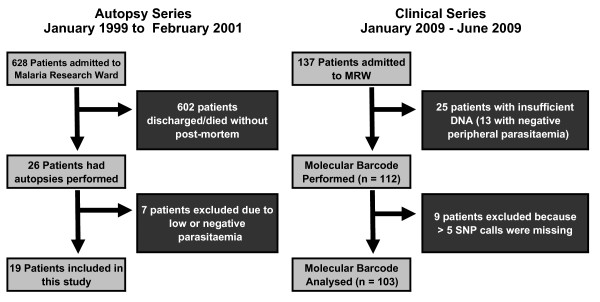
**Flowchart of patients and samples collected for these studies**.

Two cohorts of patients were included in this study, which was nested within the clinic-pathological study in the Paediatric Research Ward (PRW) of Queen Elizabeth Central Hospital in Blantyre, Malawi (Figure 1). The autopsy series includes 19 autopsies (January 1999 to June 2001) in which genotypes were previously assessed (characterized by *msp-1 *and *-2*) of tissue-sequestered parasites [5]. Tissues collected at autopsy (six sites per patient, including three brain sites, heart, lung and colon) and peripheral blood at time of admission to the research ward (one per patient when available) for the definitive pathological diagnostic groups CM vs other causes of death were compared. A total of 120 samples were available (19 × 6 tissues + 6 × 1 peripheral bloods).

The second cohort comprised all patients (n = 137) admitted between January and June of 2009. Peripheral blood was collected on FTA cards (Whatman) at admission. All genotyping was performed blinded to patient information, including parasitaemia. Two groups of patients were distinguished (i.e. those with and without malaria retinopathy) and the molecular barcodes compared in the peripheral blood between these two groups. The allele frequencies were also compared between the population of Malawi parasites in this study to the previously published collection of global isolates [4]. The research ethics committees at Michigan State University, the University of Liverpool, the University of Malawi College of Medicine, and the Brigham & Women's Hospital have approved all or appropriate portions of this study.

### The paediatric research ward

The Paediatric Research Ward (PRW) admits children, with informed parental consent, to a programme of clinical care and detailed observational studies. Patients are admitted who fulfill clinical criteria for a variety of malarial and non-malarial diagnoses. Diagnostic criteria, clinical management, laboratory investigations and treatment protocols have been previously described [18]. Final clinical diagnoses were derived from data collected throughout the hospital stay. The presence or absence of malarial retinopathy, defined as the presence in one or both eyes of vessel colour changes (orange vessels or vessel whitening), retinal whitening, and/or haemorrhages, was assessed after admission using direct and indirect ophthalmoscopy [14,15]. In the event of death, a Malawian clinician or nurse met with key family members to request their consent for an autopsy. If permission was granted, the post-mortem was performed as quickly as possible in the mortuary at the Queen Elizabeth Central Hospital (i.e. less than 12 hours).

### Autopsy procedures

Gross examination, documentation and histological assessment of the brains and other organs were performed, and a final anatomic diagnosis was determined as previously described [18]. Briefly, patients meeting the WHO clinical case definition of CM during life, who were found to have sequestration of parasites in their brain were classified as CM; patients with these features plus a haematocrit less than 15% were classified as cerebral malaria plus severe malarial anaemia (CM + SMA); patients with a haematocrit of less than 15% and no other pathology were classified as SMA; and all other patients were classified by the anatomic cause of death (e.g. pneumonia or other). Autopsy data from 19 patients were examined: five patients had CM, five had CM + SMA, one had SMA, four had pneumonia, and four had other non-malarial diagnoses.

### DNA extraction

For the 19 autopsy patients included in this study, 200 μL of peripheral blood and 0.5 g of frozen tissue from six organ sites (frontal lobe, mid-brain, cerebellum, lung, heart, colon) were used for DNA extraction by a previously described phenol:chloroform extraction protocol [5]. From the 137 admitted patients in the second part of the study, FTA peripheral blood samples were used for DNA extraction using a QIAmp DNA Blood Mini Kit (Qiagen Catalog # 51106) using three 6 mm punches.

### DNA quantification

The previously described parasite DNA quantification assay [4] was optimized for both a 96-well and a 384-well plate RT-PCR system (Broad Institute, Cambridge, MA). For the quantification of peripheral blood samples of admitted patients (Malawi-Liverpool-Wellcome Trust Clinical Research Programme (MLW), Blantyre, Malawi), where a 96-well plate system was available, a master mixture was prepared using 5.0 μl 2× Master Mix (Applied Biosystems Catalog # 4364343) and 0.5 μl of 20 × PF07_0076 pre-mixed quantification assay. Experimental internal control for standard curve (3D7 from culture in serial dilution, verified by Nanodrop quantification) and samples were loaded into 96-well PCR plates (total volume of DNA and water was 5.0 μl in a 10 μl reaction) followed by addition of the master mixture. PCR conditions and analysis were as described previously [4].

### Genotyping

The organ and peripheral blood from the autopsy patients underwent molecular barcoding using the 24-SNP assay in a 384-well format (Broad Institute, Cambridge, MA) as previously described [4]. DNA extracted from the peripheral blood samples from admitted patients underwent molecular barcoding using a 24-SNP assay in a 96-well format performed in the field (MLW, Blantyre, Malawi) as follows: template DNA and water in a total volume of 5.0 μl was added to a 5.0 μl mix made up of 0.250 μl 40× SNP assay and 4.75 μl 2× Master Mix (AB Catalog # 4364343) in a 96-well optical PCR plate and mixed, for a total reaction volume of 10 μl. The PCR amplification conditions and analytical approach were not changed [4]. For all barcodes, raw data and allelic calls were made blinded to all clinical data and independently by at least two observers (JM, DAM, JV, RD) and discrepancies were resolved by consensus.

### Molecular barcode interpretation

For each SNP call, the four possible results include: allele 1 is present; allele 2 is present; both alleles are present (heterozygous); or the assay fails. Because the parasites that are being sampled are in the intra-erythrocytic stage of their life cycle and are, therefore, haploid, identifying both alleles signifies the presence of at least two genomes. In the development of the 24-SNP molecular barcode assay, ratio experiments using known mixtures of two different single clone parasites were performed revealing the minimum ratio of individual assays. When a single allele is present, at least 90% of the DNA content being measured is from a single genome or a group of parasites sharing that allele [4]. When all 24 alleles are single calls (i.e. no heterozygous calls), this suggests a single genotype is present at the 90% or greater level. Autopsy samples were grouped by diagnosis and classified based on previous *msp-1 *and -*2 *data and the molecular barcodes (Additional file 1: Table S1).

Peripheral blood samples from the prospective clinical study were classified based solely on molecular barcode using assumptions from autopsy data. It was observed that the peripheral blood of autopsy patients did not contain more than two heterozygous calls and, in addition, that the peripheral blood signature consistently matched the tissue signature (see Results). For this analysis, zero, one, or two heterozygous calls were considered single/low complexity infections while three or more heterozygous calls were considered mixed infections. In previous work, failed reactions were always due to either absent genomic DNA (deletions/genome fragments) or insufficient DNA quantity in the sample.

### Statistical analysis

Comparisons of baseline characteristics between patients at autopsy and clinical patients with and without retinopathy were based on *t*-tests or Wilcoxon tests when studying continuous variables. The effect of date of admission on heterozygosity was explored using an over-dispersed Poisson regression. Logistic regression, unadjusted and adjusted for potential confounders, was performed to study the effect of presence of a single/low-complexity infection (as opposed to a complex population) on malaria retinopathy. Results of those models were compared to results of models that included the number of heterozygous calls, i.e. studying the impact of one unit increase in heterozygous calls on the likelihood of malaria retinopathy. Candidate confounders included patient's age, parasite density, haematocrit, and the date on which the sample was collected. These were retained in models when the P-value of their likelihood-likelihood ratio test was <0.05 or when they appreciably impacted the coefficient of the main variable of interest--less complex vs more complex population. When modelling numeric variables, linearity of associations was assessed using splines in generalized additive models. No variable had a statistically significant departure of linearity. Adding non-linear terms for the selected candidate confounders did not appreciably change the odds ratio of the main predictor of interest. All statistics and graphs were analysed and/or created with STATA v9.0 (College Station, Texas), R [19] and Graphpad Prism v4.03 (La Jolla, California). Statistical tests were considered significant to an α-level of 0.05.

## Results

### Sample collection and performance of the molecular barcode

A total of 114 tissues samples (six organs from 19 autopsies) plus six peripheral blood samples from six of the 19 autopsy patients were analysed in the retrospective study. Prospectively, 137 patients had peripheral blood collected on FTA, of whom 25 patients were excluded either because they had either insufficient DNA by quantification or because they had no evidence of peripheral parasitaemia on microscopy. For the 112 patient samples that were barcoded, a total of 2,736 individual SNP assays were performed, with 207 reaction failures (7.6%) and 466 heterozygous calls (17%); the baseline allele frequencies were different between the study population and the global collection previously studied, with one assay (SNP 14A) being non-informative in the Malawi samples (Table 1 and Additional File 2: Table S2) [4]. The molecular barcodes of 103 patients were analysed, 69 with and 34 without malaria retinopathy; nine samples were excluded owing to greater than five failed reactions out of 24. Fifty-two samples contained single/low complexity infections (zero, one, or two heterozygous calls) while 51 samples had more complex populations (≥ three heterozygous calls, maximum number observed in a sample = 19).

**Table 1:** Comparison of baseline characteristics of (A) the 19 autopsy patients presented in the first part of the study and (B) the admitted patients presenting with or without features of malaria retinopathy in the second part of the study

Characteristic	Cerebral Malaria (+/− SMA)	Non-CM (including SMA)	P*
	**(N = 10)**	**(N = 9)**	

	*Median (IQR)*		

Age [months]	24 (18-70)	25.5 (17-39)	0.64

Time [weeks]†	13.6 (9.4 - 16.4)	10 (9-14.3)	0.49

Haematocrit [%]	18 (14-31)	29.5 (22-40)	0.15

Parasitaemia [p/ul]	275,200 (11,399-572,880)	98,518 (4,716-286,850)	0.25

Characteristic	Retinopathy Positive (N = 69)	Retinopathy Negative (N = 34)	P*

	*Median (IQR)*		

Age [months]	38 (25-56)	51 (31-67)	0.19

Time [weeks]†	7.4 (4.3, 12.9)	10.4 (4.1, 17.4)	0.44

Haematocrit [%]	22 (18, 27)	30 (25, 34)	0.0003

Parasitaemia [p/ul]	101,176 (31,590-366,070)	70,230 (42,871-211,680)	0.71

### Hypothesis 1: Retrospective autopsy study shows limited parasite complexity in brain and tissue of cerebral malaria patients

**Figure 2:**
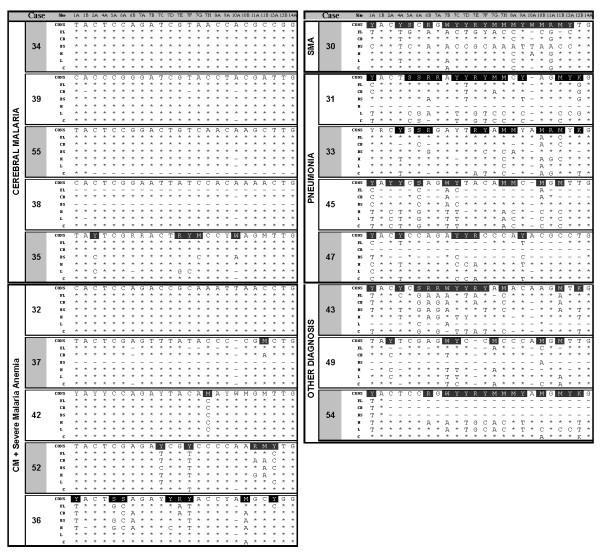
**Molecular barcoding results for 18 autopsy patients are shown with the consensus barcode in the first row and the individual barcodes for frontal lobe (FL), cerebellum (CB), brainstem (BS), heart (H), lung (L), and colon (C)**. Among the eight non-CM patients, the diagnoses were SMA (1), pneumonia (3), sepsis (1), giant cell myocarditis (1), ruptured arteriovenous malformation (1), and traumatic skull fracture (1). Where the individual barcode matches the consensus, an * is used. Where the individual barcode differs from consensus, the reported allele is given (A, G, C, or T). When the consensus barcode call is not conserved across all tissues, the consensus call is highlighted in black with white text. For those samples which were either not available or the reaction failed, a dash (−) is used to denote missing. For a heterozygous consensus call, the IUPAC code (Additional File 4) is used and BOTH bases denoted by the code are present.

Autopsy material was analysed and the genotypes of parasites sequestered in tissues and circulating in the peripheral blood were determined (Figure 1 and Table 2). The parasite molecular barcodes across tissues within an individual were nearly identical (i.e. parasites found in the heart, lungs, colon, and, when available, peripheral blood, were the same as those found in the brain - Figure 2 and Additional File 3) in patients with retinopathy-positive CM (with or without severe anaemia). It is important to note that this included some mixed infections (i.e. many heterozygous calls), primarily in the CM + SMA patients, that were still conserved across tissues, suggesting the total body population was composed of the same mixture of *P. falciparum *genetic variants. More complex infections with heterogeneity across tissues were found more commonly in the patients without evidence of retinopathy who had a range of other demonstrable causes of death. In addition, the peripheral blood reflected the extent of parasite complexity found in tissues (Additional File 3).

**Table 2:** Comparison of the major alleles encountered in the Malawi consecutive patient data set in one season (n = 112) compared with the same allele for the set of global parasites previously published (n = 114).

Assay	Malawi	Original	Difference	z-score	*p*-value
**1**	0.6446	0.8690	0.2244	-7.1029	<0.05

**2**	0.9099	0.8475	0.0625	1.8546	NS

**3**	0.6591	0.6885	0.0294	-0.6786	NS

**4**	0.8182	0.6446	0.1736	3.8716	<0.05

**5**	0.7653	0.7698	0.0045	-0.1150	NS

**6**	0.6269	0.6154	0.0115	0.2520	NS

**7**	0.6270	0.4640	0.1630	3.4894	<0.05

**8**	0.8130	0.8034	0.0096	0.2576	NS

**9**	0.7481	0.5873	0.1608	3.4871	<0.05

**10**	0.6391	0.6111	0.0280	0.6130	NS

**11**	0.6529	0.3220	0.3309	7.5603	<0.05

**12**	0.5620	0.4274	0.1346	2.9058	<0.05

**13**	0.6504	0.5289	0.1215	2.5985	<0.05

**14**	0.7241	0.6239	0.1002	2.2087	<0.05

**15**	0.5489	0.5124	0.0365	0.7791	NS

**16**	0.7652	0.4872	0.2780	5.9392	<0.05

**17**	0.5556	0.6032	0.0476	-1.0392	NS

**18**	0.6330	0.5124	0.1206	2.5768	<0.05

**19**	0.5909	0.6250	0.0341	-0.7519	NS

**20**	0.6692	0.6230	0.0462	1.0183	NS

**21**	0.5231	0.4318	0.0913	1.9671	<0.05

**22**	0.5766	0.3953	0.1813	3.9591	<0.05

**23**	0.6866	0.6518	0.0348	0.7795	NS

**24**	0.9231	0.5897	0.3333	7.2356	<0.05

Thirteen assays are significantly different from the population observed in the original study with the differences ranging from 9.1 to 41.0%. Assay 24 is non-informative in the Malawi population

### Hypothesis 2: Prospective clinical study shows limited parasite complexity in peripheral blood of patients with cerebral malaria

**Figure 3:**
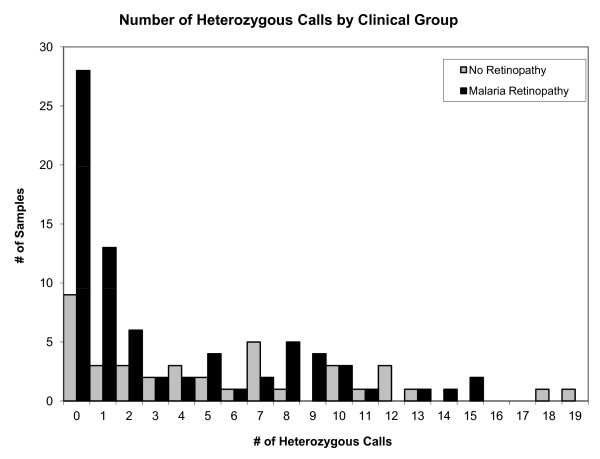
**Molecular barcodes were grouped by number of heterozygous calls (range = 0-19) after excluding barcodes missing in more than five assays and were then categorized by malaria retinopathy (presence or absence)**. A one unit decrease in heterozygous calls is associated with a increased risk of presenting with malaria retinopathy: adjusted OR 1.11; 95% CI = 1.01, 1.22; P = 0.03.

When comparing retinopathy-positive to retinopathy-negative clinical patients, 77% of retinopathy-positive patients had single/low complexity infections including 28 samples with zero heterozygous calls suggesting a single genotype (Figure 3). The unadjusted OR for having positive malarial retinopathy in individuals with a low complex infection was 3.70. A sensitivity analysis was performed using only molecular barcodes with one failure, etc. up to five failures within an individual barcode and the same unadjusted odds ratio was calculated (3.7-4.2), which was significant (*P*-value < 0.01). Patients with and without malaria retinopathy were clinically similar except for haematocrit (retinopathy-positive < < retinopathy-negative; Table 2). When adjusted for haematocrit, parasite density (confounding variable), and the effect of time, the OR for a single/less complex (ci more complex) infection having retinopathy was 4.8 (Table 3).

**Table 3:** Association between malaria retinopathy and infections caused by a single/less complex genotypes (vs three or more).

	Single/Less Complex Genotype	Multiple Genotypes	
	
	(< 3 Het. Calls)	(≥3 Het. Calls)	
	**(N = 52)**	**(N = 51)**	

**Retinopathy**	42 (81%)	27 (53%)	

**No retinopathy**	10	24	

	**Logistic Regression**		
	
	**(N = 67 with and 32 without retinopathy)**		

**Unadjusted Model†**	**OR**	**(95% CI)**	P*

Single/Less Complex (vs. 3 Hets)	3.7	(1.51-9.10)	0.003

**Adjusted Model†**			

Single/Less Complex (vs. ≥ 3 Hets)	4.82	(1.59-14.30)	0.003

Hematocrit	0.84	(0.79, 0.96)	< 0.0001

Parasites/μl (natural logarithm)	1.36	(0.996, 1.85)	0.046

Time (weeks from January until date of sample collection)	0.87	(0.77-0.92)	0.001

Frequencies and odds ratios (OR) obtained in logistic regression models. Four patients were removed from regression modeling due to missing data

*Het*. = Heterozygous; *OR *= Odd Ratio; *CI *= Confidence Interval

*P-values were estimated using likelihood-ratio tests.

**†**c-statistic (corresponding to the area under the ROC curve of the fitted model) of the unadjusted model was 0.66 and of the adjusted model was 0.82.

### Hypothesis 3: There is not a single genotype that causes cerebral malaria

Despite the preponderance of single/less complex parasite populations in cerebral malaria patients, there was no unique genotype or group of genotypes associated with cerebral malaria in different patients.

### Observation: The genetic complexity of infections decreased over the malaria season

**Figure 4:**
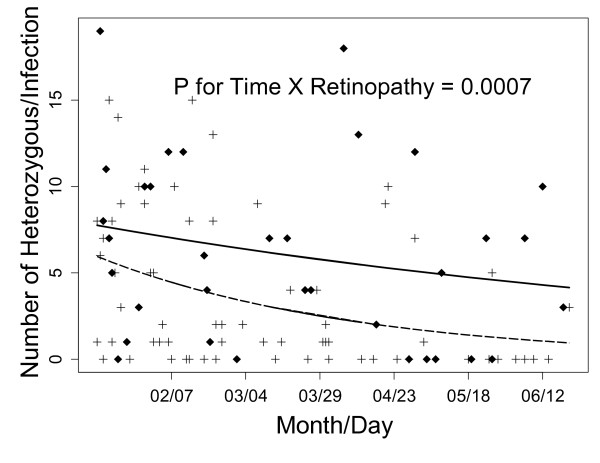
**Changes in the number of heterozygous calls per barcode in individual patients (Y-axis) as days of sample collection progressed (X-axis) in the malaria season of 2009 (January to June 2009)**. The crosses and dashed line (obtained from an over-dispersed Poisson model) represent the malaria retinopathy-positive patients with a decrease of approximately 5.0 heterozygous calls over six months. The diamonds and solid line (obtained in an over-dispersed Poisson model) represent the malaria retinopathy-negative patients with a less marked decrease of approximately 3.6 heterozygous calls over six months. The trend suggests that over the course of the data collection period (i.e. the malaria season in Malawi), the number of mixed infections decreases leading to more homogenous infections. Trends over time of patients with and without malaria retinopathy were statistically significantly different (*P*-value = 0.0007).

The complexity of infections in both patient groups decreased over the six months during which data were collected (i.e. from start to end of the malaria season). Retinopathy-positive CM patients had consistently and significantly fewer heterozygous calls per patient (*p*-value = 0.00007, Figure 4) than retinopathy-negative patients, throughout the period of study.

## Discussion

Malaria parasites cause more than 200 million clinical infections per year with 800,000 deaths, due primarily to *P. falciparum *in sub-Saharan African children [12,20]. Paediatric malaria mortality is largely restricted to non- and semi-immune children, and commonly results from cerebral malaria (CM) [21]. The clinical diagnosis of CM is strongly associated with the pathological finding of parasite sequestration (attributable to cytoadherence of parasites to endothelium) within the cerebral vasculature [18,22]. The definitive diagnosis of CM relies on post-mortem examination of the brain either by autopsy or supra-orbital sampling [13]; yet, the pathogenesis of CM and etiology of death in fatal cases remain poorly understood.

Genotyping using *msp-1 *and -*2 *(or any polymorphic locus by PCR without quantification) provides an accurate count of the number of genotypes present in a sample. Because the majority of these assays are nested PCR reactions, they can detect very small amounts of any contributing genome; however, the relative contributions of a given genome cannot be determined without specialized secondary techniques (such as capillary electrophoresis). Previous work using standard *msp-1 *and -*2 *genotyping has shown that patients who have an acute malaria illness (i.e. who present with symptoms and peripheral parasitaemia) of any degree of severity tend to have genetically less complex infections than asymptomatic patients screened in the same population [7,10,11]. When quantitative methods are applied to *msp-1 *and -*2 *using capillary electrophoresis and fluorescent tags, patients presenting with acute malaria illness have been shown to present with a dominant *msp *allele (as determined by relative quantities of individual *msp *alleles) [8,9]. These two observations, taken together, suggest that patients with an acute malaria illness are often ill with dominant genotypes (i.e. a single or small set of similar genotypes composing the largest proportion of the total circulating parasite biomass). This study focused exclusively on children who meet the clinical case definition of cerebral malaria, all of whom had *P. falciparum *parasitaemia. Patients were separated solely on the presence (retinopathy-positive CM) or absence (retinopathy-negative CM) of malaria retinopathy to ask what the differences are in genetic components of infection between these two phenotypes using the molecular barcode technique. Genotyping by *msp-1 *and -*2 *has been used to evaluate these patients previously [5] and the current study supports and extends those observations to show a dominant genotype by barcoding.

Previous studies have shown that malaria retinopathy is strongly associated with pathological confirmation of cerebral parasite sequestration, while parasitaemic comatose patients without malaria retinopathy represent a diverse group of non-malarial diseases [17,18]. This current data support the concept of limited parasite diversity in cerebral malaria patients and are consistent with mathematical models and immunological evidence from a broad range of studies [23-34]. These results validate the previous *msp-1 *and *msp-2 *data; importantly, they demonstrate that the molecular barcodes from peripheral parasitaemia mirror those generated from sequestered parasites in various tissues and this genotype is predominant in CM [5]. Histological observations in children dying of non-malaria causes have demonstrated a paucity of sequestration in the many tissues examined in such patients (unpublished data, manuscript pending) [5,35]. By contrast, patients dying of cerebral malaria have dense parasite sequestration throughout the body on histological examination [5,35]. The study was designed assuming that parasites found in the peripheral blood of retinopathy-negative patients are likely to be incidental to the patient's illness and are not reflective of a dense sequestered mass of parasites (in contrast to the situation in patients with retinopathy).

The number of heterozygous calls within individual patients with retinopathy-positive CM decreased over the malaria season, suggesting that complexity of infection is a product of time. This could be explained by a decreasing rate of parasite inoculations, or an increasingly broad acquisition of immunity in the population as the season progresses. Clinically evident infections may, as immunity accumulates, tend to occur only in patients inoculated with new genotypes for which no immunity has developed.

Parasites from peripheral blood and parasites found in tissues at autopsy had the same conserved barcode identification within an individual patient in CM patients. Less complex infections in the peripheral blood were more common in patients with stringently characterized cerebral malaria (standard clinical case definition plus retinal examination) and include apparently single genotypes fairly frequently (35%). However, there was not a single global conserved barcode genotype associated with cerebral malaria. The tissues of patients without cerebral malaria showed different signals in different tissues, and this heterogeneity is reflected in the peripheral blood samples. The corresponding histological tissue slides from these patients show very few sequestered parasites suggesting that the signal from individual tissues came from either small collections of sequestered parasites and/or parasite DNA within phagocytic macrophages. Cerebral malaria patients had less complex infections in tissue (average of 1.6 heterozygous calls per sample = two to three genotypes/sample), a finding consistent with the earlier study in which the complexity was characterized using *msp-1 *and *msp-2 *genotyping (average of 1.9 genotypes per sample) and with the literature (Additional file 1: Table S1) [5].

What remains unknown is the full range of diversity within a single cerebral malaria patient with such an enormous sequestered burden. The autopsy data presented here are from patients who died from the illness; thus, single/low complexity infections may be more likely to result in death. The observations made in this clinical cohort suggest that a wider range of diversity is possible within the individual with or without retinopathy, although in these clinical patients those with retinopathy had significantly less complex infections than those without.

## Conclusions

Using a novel molecular barcode assay, previous findings were confirmed that in patients with various syndromes associated with *P. falciparum *infection, the genetic complexity of the parasite population is lowest in CM, intermediate in CM combined with severe anaemia, and highest in subjects with incidental parasitaemia. The complexity of *P. falciparum *infections as measured in peripheral blood decreases during the malaria season in all patient groups, being always lowest in CM. Most patients with CM harbour a parasite population dominated by a single genetic variant that is identical throughout both the blood and organ sites, but the predominating variant differs between patients. Parasites retrieved from peripheral blood have a genetic composition similar to those sequestered in tissues. Peripheral blood is more readily available than deep tissues and, unlike deep tissue, can be obtained from patients with non-fatal as well as fatal malaria, allowing for very large collections of patient data sets. Techniques have recently become available for sequence analysis directly from patients that not only identify but quantify genome contributions. Data from this study support the use of peripheral blood samples to study the contribution of parasite genetics to the clinical syndrome of cerebral malaria.

## Competing interests

The authors declare that they have no competing interests.

## Authors' contributions

DAM designed the study, performed the experiments and statistical analysis, and wrote the manuscript. JV, JM, NCM, KGB, DMR performed the experiments. CV performed the statistical analysis. RFD, SKV, DEN, DJP, SFS, AKL, DVT, RCW, and PCS designed, developed, and validated the barcoding assay. KBS, SJG, SK, MEM, and TET provided clinical care and data collection for all patients. DFW designed the study and edited the manuscript. All authors read and approved the final manuscript.

## Supplementary Material

Additional file 1 **Comparison of the number of msp-1 and -*msp-*2 alleles found in peripheral blood and tissues of patients with different diagnosis with the number of heterozygous calls found using the molecular barcode demonstrates low complexity in cerebral malaria**. Statistical comparison was made by ANOVA. Comparison of the average number of heterozygous calls found using the molecular barcode in the clinical peripheral blood samples shows a significant difference between retinopathy positive and retinopathy negative. The slightly higher average value of the retinopathy positives is due to the inclusion of patients who may be CM and SMA. Statistical comparison was made by *t*-test of means. Click here for file

Additional file 2 **Table S2**. The barcodes for 112 consecutive patients with positive peripheral blood parasitaemia and sufficient DNA for performance of the molecular barcode are shown. The order of the patients displayed in the table is by study number and not by date (see Figure 4). Click here for file

Additional file 3 **The complete molecular barcodes (24 TaqMan SNP Assay) for 19 patients who had pathological evidence of cerebral malaria at autopsy**. Peripheral blood DNA at the time of admission was available from five CM patients and three non-CM patients and generally showed the same genotype as tissues, with more variation in the CM + SMA patients and non-CM patients. Sample types include peripheral blood (PB), frontal lobe (FB), cerebellum (CB), brainstem (BS), heart (H), lung (L), and colon (C). The major alleles are presented in a white background and the minor alleles are presented with a grey background. The frequency of major and minor alleles for the population of Malawi parasites examined in this study was significantly different from those seen in the global population (Table 2). For a heterozygous call, the IUPAC code is used and BOTH bases denoted by the code are present (orange/light-grey background). Failed reactions are shown as "X". See Additional File 4 for IUPAC codes used in this context. Click here for file

Additional file 4 **Descriptions of the assays used in this study are shown including the derived major and minor allele frequencies for the Malawi data set as well as codes for heterozygous allele calls (both alleles present) in the standard IUPAC format**. For a complete list of chromosome positions, primer sequences, and probe sequences, please see Additional File 3 in previous publication ([http://www.biomedcentral.com/content/supplementary/1475-2875-7-223- S3.pdf]). Click here for file
